# Exploring and modeling recurrent birth events in Ethiopia: EMDHS 2019

**DOI:** 10.1186/s12884-022-04948-w

**Published:** 2022-08-05

**Authors:** Lijalem Melie Tesfaw, Essey Kebede Muluneh

**Affiliations:** 1 Department of Statistics, Bahir Dar University, Bahir Dar, Ethiopia; 2 School of Public Health, Bahir Dar University, Bahir Dar, Ethiopia

**Keywords:** Andersen-Gill and Frailty model, Ethiopia, Recurrent birth, Prentice, Prentice, Williams, and Peterson total time model, Prentice, Williams, and Peterson gap-time model

## Abstract

**Background:**

Globally, the estimated recurrent childbirth of one forth of women happens every two years or fewer. Next to Nigeria, Ethiopia is the second populist country in Africa and the first populist country in sub-Saharan Africa which consists of over 114 million population. There are prevalent short successive birth intervals problems in underdeveloped nations such as Ethiopia that contributes to adverse effects on mother and child health. However, studies that explore recurrent birth events and associated factors are very limited. Thus, this study aimed to explore and model the recurrent birth event by considering its subsequent within a mother and identifying its important determinants. As a result, the research findings of this study will be one of the preliminary research papers on the recurrent birth events that take into account the subsequent event and enable to be input for the policymakers, health institutions, and future researchers.

**Methods:**

A total of 4676 mothers with their 16833 corresponding children were involved in this study. The data was obtained from the 2019 Ethiopian Mini Demographic and Health Survey (EMDHS). In this study, extended cox regression models such as Andersen-Gill (AG), Prentice, Williams, and Peterson total time (PWP-TT) model, PWP-gap time (PWP-GT) model, and Frailty model were employed. These all models are used to consider recurrent events within mothers and determine the potential determinants. AG, PWP-TT, and PWP-GT estimate the effect of covariates by considering the correlation between event times for a person can be explained by past events given the covariates, k^th^ event since the entry time, k^th^ event since the time from the previous event, respectively.

**Results:**

Among mothers who have first and ninth recurrent birth events, 75.5% and 80.1% of them respectively were rural residents while 57.6% and 70.6% of them respectively were a place of delivery in the health sector. The highest prevalence of first recurrent births (44.3%) was obtained from Muslim mothers. Mothers' education level (HR: 1.210; 95%CI: 1.010, 1.460), mothers' age at first birth (HR: 0.713; 95% CI: 0.614, 0.828), household wealth index (HR: 0.776; 95% CI: 0.625, 0.965), child mortality (HR: 0.673; 95%CI: 0.514, 0.881), household size (HR: 1.914; 95%CI:1.539,2.381) and sex of child (HR:0.836; 95%CI = 0.755,0.926) were important determinants of recurrent birth event. This indicates mothers’ education level and household size were positively associated with recurrent birth events. Whereas mothers’ age at first birth, household wealth index, child mortality, and sex of the child was negatively associated with recurrent birth events.

**Conclusion:**

The WHO recommends a minimum of 33 months between two consecutive births, which is longer than the Ethiopian recurrent birth intervals observed in this study. The highest recurrent birth occurred during the age of fewer than twenty years old of mothers at first birth as compared to mothers whose age was older at first birth. Mothers, children, and household characteristics had significant effects on recurrent birth events. We authors would like to recommend communities, governmental and non-governmental stakeholders consider the associated factors of frequent recurrence of birth noticed in this study. Besides, we would also like to recommend women start birth while they got mature in age to reduce frequent recurrent birth and its corresponding adverse effects.

## Background

Recurrent birth is defined by the WHO as two or more consecutive births of women [1]. Globally, the estimated recurrent childbirth of one forth of women happens every two years or fewer. Most of the short succussive birth intervals were noticed from Central Asia and Sub-Saharan Africa. It helps to explain why the fertility rate is so high. The world's population is growing at an alarming rate. Developing countries, such as Africa, are responsible for the majority of the population increase [1, 2]. In Sub-Saharan Africa, where the population is expected to more than quadruple in the next 40 years, high fertility and recurrent births are driving population increase [1, 3].

According to United Nations (UN) projections from 2019, Sub-Saharan Africa's total reproduction rate, at 4.7 births per woman in 2015–2020, is more than double that of any other world area. As a result, Africa's population is predicted to increase from 1 billion in 2015 to over 2 billion in 2050 and approximately 4 billion in 2100. This upward population expansion causes the economy to become incompatible with the population, even for fundamental requirements. This has a variety of negative consequences for human well-being and the environment [2]. There are several socioeconomic, demographic, biological, and behavioral factors such as the desired number of children in the family and religions associated with fertility control [1, 3].

Next to Nigeria, Ethiopia is the second populist country in Africa and the first populist country in sub-Saharan Africa which consists of over 114 million population [3, 4]. Behavioral and biological factors, as well as demographic factors, are background drivers of women's recurrent birth, according to a study done in Ethiopia in 2019. These factors modulate the influence of culture, society, economic situations, and living standards [3]. Two or more successive childbirths of women constitute a recurrent birth occurrence in the sense that the delivery of one or more children within a certain time frame, from live birth to the following birth. Women are more vulnerable to macro and micronutrient depletion during pregnancy and breastfeeding when the periods between recurrent deliveries are shorter (less than or equal to 24 months). This causes problems with later pregnancies and child health [5]. Commonly the successive time interval of the recurrent birth is measured in terms of years [4, 6]. Short-term recurrent birth in the family is highly responsible for the mortality and morbidity of a child. In developing countries, the years' gap between children at successive births is short and leads to inadequacy of food mainly breastfeeding [5]. It is a prevalent problem in underdeveloped nations such as Ethiopia, and it contributes significantly to poor mother and child health [2, 4].

Studies [1, 2, 7] reported that residence area, husbands’ occupation, type of contraceptive used, and sex of preceding children highly determined the duration between successive births. Traditionally, mothers believe that the use of contraceptives for birth spacing can be affected by son preference where the last child born is a female. Besides, mothers significantly reduced birth intervals in the quest for a male child when more female children [5, 7]. According to a demographic and health survey (DHS) report higher than ten percent of the child recurrent births in African countries occurred within less than twenty-four months. The proportion of recurrent birth within less than 24 years in Tanzania, Nigeria, Zimbabwe, and Kenya was 19, 23, 11, and 18% respectively [5].

A study in Uganda revealed that most of the short recurrent birth intervals occurred among rural women as compared to women in the urban area. The study also reported that the younger maternal age and lack of husband influence on the duration of the next baby are the most influential factors [5]. Beyond this, maternal and husband education, household wealth index, religion, breastfeeding duration, and maternal age are important determinants of the time elapsed between two consecutive live births [4]. A study in [8] indicates that more than half of women in Ethiopia have a shorter birth interval that links with child mortality and morbidity as well as mothers' adverse health effects.

Though numerous studies on overall fertility, as far as the researchers’ knowledge is concerned there is very limited study on the recurrent birth events in Africa and Ethiopia in particular. Sometimes the event of interest can occur more than once in a subject the so-called recurrent events. In our case recurrent birth events. However, several studies in [9–11] focus on time to the first events and apply a cross-sectional study design, ignoring the subsequent event which is not taken into account the correlation between recurrent birth events within a mother. Thus, this study aimed to address this limitation by exploring and modeling the recurrent birth events by considering their subsequent within a mother and identifying the important determinants. Besides, the study also aimed to demonstrate the updated recurrent birth events practiced and family planning/size in Ethiopia. Due to this, the research findings of this study will be one of the preliminary research papers on recurrent birth events that take into account the subsequent event and enable to be input for the future researcher, policymakers, and health institutions.

## Methods

### Data source

In this study, a total of 4676 women who have delivered at least one live birth were considered. The data was obtained from the 2019 EMDHS. This secondary data was downloaded from the DHS website www.dhsprogram.com. Providing up-to-date estimates of key demographic and health indicators by collecting high-quality data for maternal and child health are the primary objectives of EDHS among its multidisciplinary objectives [12]. The sampling frame used for the 2019 EMDHS is a frame of all census enumeration areas (EAs) created for the 2019 Ethiopia Population and Housing Census (EPHC) and conducted by the Central Statistical Agency (CSA). The Ethiopian Public Health Institute (EPHI), in collaboration with the Central Statistical Agency (CSA) and the Federal Ministry of Health (FMOH), implemented the EMDHS 2019 under the overall leadership of the Technical Working Group (TWG). The World Bank, the United States Agency for International Development (USAID), and the United Nations Children's Fund (UNICEF) all contributed to the 2019 EMDHS. The 2019 EMDHS sample was stratified and selected in two stages. In the first stage, a total of 305 EAs were selected with probability proportional to EA size and with independent selection in each sampling stratum. A household listing operation was carried out in all selected EAs from January through April 2019. The resulting lists of households served as a sampling frame for the selection of households in the second stage. In the second stage of selection, a fixed number of 30 households per cluster were selected with an equal probability of systematic selection from the newly created household listing, and maternal as well as children characteristics were obtained via interview and questionnaire [12].

### Data description

The sample data consists of mothers' identification number, children's birth order, start and stop time of birth, event, and gaps (in years) revealed in Table 1. Besides, additional mothers, household, and child characteristics are also presented in Table 2.

**Table 1:** Sample data format of mothers and children birth order

Mother’s ID	Children birth order	Start time	End time	Event	Gap (in years)
0001	1	0	1.8	1	1.8
0001	2	1.8	3.0	1	1.2
0001	3	3.0	4.5	1	1.5
0002	1	0	3.2	1	3.2
0002	2	3.2	5.0	1	1.8
.	.	.	.	.	.
.	.	.	.	.	.
.	.	.	.	.	.
4675	1	0	3	0	3.0
4676	1	0	1.7	1	1.7
4676	2	1.7	2.9	1	1.2
4676	3	2.3	5.0	1	2.7
4676	4	5.0	7.2	1	1.2

**Table 2:** Mothers, household, and child characteristics description

Variables	Categories (Codes)	Weighted frequency
**Mothers characteristics**		*n* = 4676 (%)
Age of mothers at first birth	< 20 (1)	3289 (70.3)
	20–34 (2)	1379 (29.5)
	35–49 (3)	8 (0.2)
Mother’s current marital status	Married (1)	4159 (88.9)
	Widowed (2)	198 (4.2)
	Divorced (3)	319 (6.8)
Place of delivery	Home (1)	1605 (34.3)
	Health sector (2)	1507 (32.2)
	Missing	1564 (33.4)
Contraceptive use	Modern (1)	1396 (29.9)
	Traditional (2)	37 (0.8)
	Not Using (3)	3243 (69.4)
Mother’s education level	No education (0)	2890 (61.8)
	Primary (1)	1281 (27.4)
	Secondary and above (2)	505 (10.8)
Husband/partner’s education level	Primary (1)	2216 (47.4)
	Secondary and above (2)	1487 (31.8)
	Missing	973 (20.8)
**Household characteristics**		*n* = 4676(%)
Residence	Urban (1)	1148 (24.6)
	Rural (2)	3528 (75.4)
Region	Tigray (1)	386 (8.3)
	Afar (2)	408 (8.7)
	Amhara (3)	519 (11.1)
	Oromia (4)	594 (12.7)
	Somali (5)	391 (8.4)
	Benishangul (6)	425 (9.1)
	SNNPR (7)	606 (13.0)
	Gambela (8)	413 (8.8)
	Harari (9)	366 (7.8)
	Addis Ababa (10)	230 (4.9)
	Dire Dawa (11)	338 (7.2)
Religion	Orthodox (1)	1538 (32.9)
	Protestant (2)	959 (20.5)
	Muslim (3)	2076 (44.4)
	Catholic, traditional, and others (4)	103 (2.2)
Household size	1–4 (small) (0)	1250 (26.7)
	5–9 (medium) (1)	3165 (67.7)
	10 and more (Large) (2)	261 (5.6)
Household wealth index	Poorest (0)	1332 (28.5)
	Poorer (1)	816 (17.5)
	Middle (2)	754 (16.1)
	Richer (3)	726 (15.5)
	Richest (4)	1048 (22.4)
**Child characteristic**s		*n* = 16,833 ( %)
Sex of child	Male (1)	8624 (51.2)
	Female (2)	8209 (48.8)
Child is alive	Yes (1)	15,230 (90.5)
	No (2)	1603 (9.5)

**Fig. 1:**
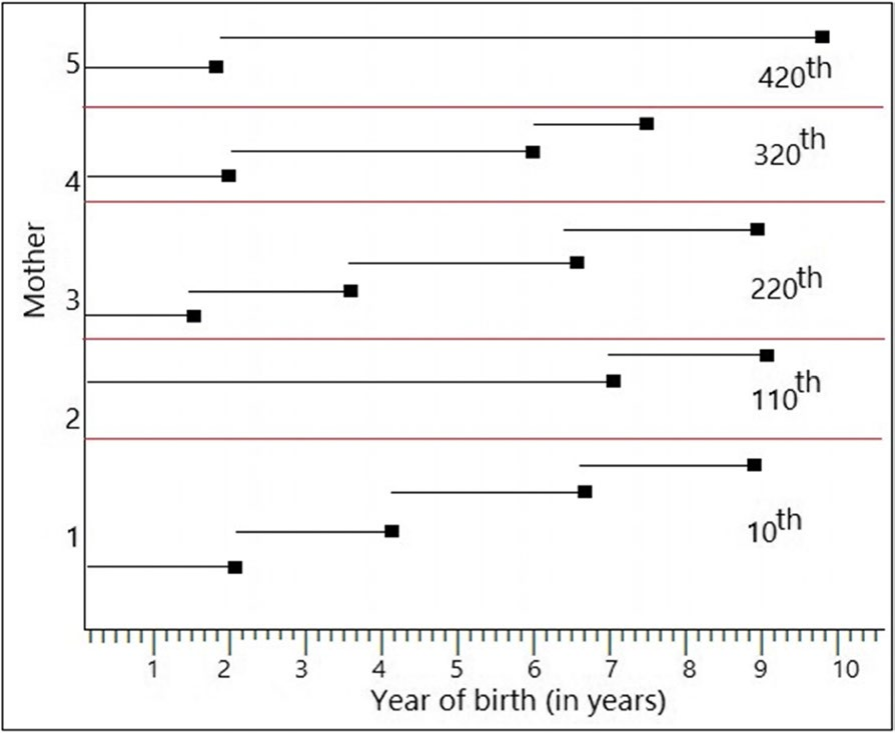
Recurrent birth events of children for a sample of five mothers

A sample of five mothers (10^th^, 110^th^, 220^th^, 320^th^, and 420^th^) with their corresponding recurrent birth and duration of birth (in years) was depicted in Fig. 1. For instance, the first mother has four recurrent births and the duration of birth for the first, second, third, and fourth births were 2.1, 2.0, 2.7, and 2.2 years respectively. Similarly, the second, third, fourth, and fifth mothers had two, four, three, and two recurrent births with different duration of the birth.

### Inclusion/exclusion criteria

As the study is on the recurrent birth event, only women who have at least one recurrent birth were eligible for this study.

### Variables

The dependent and independent variables considered in this study were presented in Table 2. The recurrent birth event is the dependent variable of the study. The recurrent birth event is defined as two or more successive deliveries [5]. Further justification of the recurrent birth was also presented in Tables 1 and 3. Mothers, household, and child characteristics presented in Table 2 were considered independent variables. The selection of independent variables is theoretically driven that draws support from prior research concerning factors affecting time elapsing to successive deliveries [4].

**Table 3:** Summary statistics of the recurrent birth event

		Duration of recurrent birth (in years)			
Event	Frequency (%)	Minimum	Maximum	Median time	Std.deviation
1st recurrent	4632 (27.5)	1.20	17.9	3.00	2.10
2nd recurrent	3600 (21.4)	1.00	18.0	3.00	2.00
3rd recurrent	2802 (16.6)	1.20	15.0	2.00	1.87
4th recurrent	2084 (12.4)	0.95	19.0	2.00	1.82
5th recurrent	1483 (8.8)	1.70	15.2	2.00	1.65
6th recurrent	983 (5.8)	1.30	12.8	2.00	1.65
7th recurrent	584 (3.5)	1.80	15.9	2.00	1.68
8th recurrent	334 (2.0)	1.50	7.9	2.00	1.40
9th recurrent	176 (1.0)	1.30	9.0	2.00	1.34
10th recurrent	92 (0.5)	1.70	7.0	2.00	1.30
11th recurrent	38 (0.2)	1.50	11.5	2.00	2.23
12th recurrent	14 (0.1)	0.95	7.0	2.00	1.70
13th recurrent	9 (0.1)	1.10	4.0	2.00	1.00
14th recurrent	2 (0.0)	0.95	4.0	2.50	2.12

### Statistical analysis

The standard cox proportional hazard model is used to estimate the effects of determinants on time to event outcome of interest assuming that each of the observations is independent. In this study, the recurrent birth events were measured from each mother. From this fact, we can deduce the presence of a non-ignorable correlation between recurrent birth events of the same mother. As a result, implementing the standard cox proportional hazard model may end in a biased estimate. This problem can be addressed by using the extended cox regression models that take into account the correlation between recurrent birth events within a mother [13]. The most common extended cox regression models are Andersen-Gill (AG), Prentice, Williams, and Peterson (PWP), Wei, Lin, and Weissfeld (WLW), and frailty models.

### Andersen-Gill (AG) Model

AG model is formulated in terms of increments in the number of events along the timeline. The outcome of interest is time until an event occurs or time since study entry also known as the total time scale [13]. It uses a common baseline hazard function for all events and estimates a global parameter for the factors of interest. The AG model assumes that the correlation between event times for a person can be explained by past events given the covariates. The counting process style of data input is seen in the AG model where each subject is represented as a series of observations with recurrence time given as (t0, t1], (t1, t2] … (tm, last follow-up time] where each recurrent event for the ith subject is assumed to follow a proportional hazard model is given as [13, 14]

1 $$\lambda i(t) = \lambda 0(t)exp(\beta kXi(t))$$

where *β*_*k*_ is the estimated parameters of the corresponding *X*_*i*(*t*)_ covariates.

### Prentice, Williams and Peterson (PWP) Model

The PWP model analyses ordered multiple events by stratification, based on the prior number of events. The stratification PWP models are i) PWP total time (PWP-TT), which evaluates the effect of a covariate for the kth event since the entry time in the study; ii) PWP gap time (PWP-GT), which evaluates the effect of a covariate for the k^th^ event since the time from the previous event. Both PWP approaches are conditional models as an individual is not considered in the risk set for the k^th^ event until experiencing the (k − 1)^th^ event. The baseline hazards vary from event to event, the hazard function for the kth event for the ith subject with the PH of PWP-TT model is given by:

2 $$\lambda ik(t) = \lambda 0(t)exp(\beta kXi(t))$$

The PWP—GT model describes an intensity process from the occurrence of an immediately preceding event, with the gap time defined as (t-t_*k*−1_).

3 $$\lambda ik(t) = \lambda 0(t-tk-1)exp(\beta kXi(t))$$

where *λ*_0*k*(*t*)_ is the common baseline hazard function.

### Frailty model

In survival analysis, the model that enables account for random effects is called the frailty model [13, 15]. The dependence on the recurrent event time can be induced using the frailty model. The model assumes that the recurrent event times are conditional on the covariates and random effects. This study diversified individuals with different hazards, but the characteristics for differences between individuals are not captured by the measured covariates. In such applications, frailty models can be a possible choice [13]. Thus, during the analysis using the frailty model the correlation between recurrent birth events within a mother was taken under consideration by considering the random effects.

**Fig. 2:**
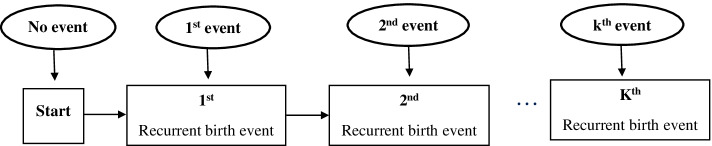
Framework of recurrent birth events

In the framework of recurrent birth events in Fig. 2, the boxes indicate the states while the arrow between states indicates the transitions or changes of states. The k recurrent events revealed in Fig. 2 indicate the number of events that occurred per participant. For instance, in this study, the maximum number of recurrent birth events was 14, see Table 2.

### Hazard ratio

In survival analysis, the hazard ratio is used to interpret the association/effect of exposures on the hazard of an event. It is the ratio of two hazards. A hazard ratio of one indicates a lack of association, a hazard ratio greater than one suggests an increased risk, and a hazard ratio below one suggests a smaller risk. Hazard ratios of interest are derived using the regression coefficient of the extended Cox models and reflect a comparison of two different sets of covariate values. For instance, in our case, if the covariate X_*i*_ is status for a particular risk factor, then the hazard ratio is obtained by the exponentiation of the estimated regression coefficient for X_*i*_, exp{*β*_*i*_}.

The data organization was done using SPSS 25 while the descriptive and inferential statistical analysis was done using R software version 3.4.

### Ethics approval and consent to participate

Since the study was a secondary data analysis of publicly available survey data from the measure DHS program, ethical approval and participant consent were not necessary for this particular study. We requested DHS Program and permission was granted to download and use the data for this study from www.dhsprogram.com. Permission was obtained to use the EDHS data from the Measure DHS international program. The data is publicly available and has no personal identifiers. We confirm that all methods were carried out following the relevant guidelines and regulations.

## Results

The maternal, child, and household characteristics description and frequency distribution were presented in Table 2. A total of 4676 mothers and their 16833 respective children were involved in the study. The majority of mothers (69.8%) had no education while the majority of husbands/partners (47.4%) had a primary education level. The proportion of mothers whose place of delivery was at home (34.3%) was almost closed to the proportion of mothers whose place of delivery was in the health sector (32.2%). The proportion of mothers whose age was less than 20 years at their first birth was over seventeen percent (70.3%), an indicator that early marriage in Ethiopia continues. The highest proportion of households was living in rural areas (75.4%) and from the SNNPR region(13.0%). Almost a quarter of the household were Muslim (20.5%) while most of the households has a family size between five and nine (67.7%) and the poorest wealth index(28.5%). Of the total of 16833 children obtained from 4676 mothers, 51.2% of them were male and 90.5% were alive.

Mothers’ first delivery is considered as the time origin and the second delivery is considered as the first recurrent event, see Fig. 1. In this manner, the third, fourth, fifth, sixth delivery, etc. are the second, third, fourth, and fifth recurrent events respectively. The maximum recurrent birth event was 14 in the sense that a mother delivered 15 children, see Table 3. The median follow-up time for 3rd, 4th, 5th, 6th, 7th, 8th, 9th, 10th, 11th, 12th, and 13th recurrent birth was two years. Whereas, the median follow-up time to first and second recurrent birth was three years and the median follow-up time to fourteenth recurrent birth was two and half years. In Table 3, the minimum and maximum duration of birth (in years) of each recurrent birth event were also depicted. For instance, the minimum and maximum duration of the first recurrent birth were 1.20 and 17.9 years respectively. The minimum duration of birth (0.95 years) from 4^th^, 12^th^, and 14^th^ recurrent birth events indicate a women successive birth within a gap of less than a year.

The standard deviation revealed the average median time deviation from each duration of time (in years) of mothers in each recurrent birth.

Table 4 shows the frequency distribution of mothers, households, and child characteristics over recurrent birth events. Besides, the association between each characteristic and the recurrent birth events was evaluated. Age of mothers at first birth, mother’s current marital status, place of delivery, residence, mothers and husband/partner education level, region, religion, household size, household wealth index, and child death was independently associated with recurrent birth events. Among the total of 4632 mothers who have first recurrent birth, the age of 70.5% of them was less than twenty years old at their first birth.

**Table 4:** Independent variables description across recurrent birth event

**Variables**				**Recurrent birth events**					
	**1st**	**2nd**	**3rd**	**4th**	**5th**	**6th**	**7th**	**8th**	** ≥ 9 th**
	n (%)	n (%)	n (%)	n (%)	n (%)	n (%)	n (%)	n (%)	n ( %)
**Age of mothers at first birth**									
< 20*	3264(70.5)	2673(74.3)	2138(76.3)	1644(78.9)	1194(80.5)	815(82.9)	495(84.8)	284(85.0)	273(82.5)
20–34	1361(29.4)	922(25.6)	662(23.6)	439(21.1)	288(19.4)	168(17.1)	89(15.2)	50(15.0)	58(17.5)
35–49	7(0.2)	5(0.1)	2(0.1)	1(0.1)	1(0.1)	0(0.0)	0(0.0)	0(0.0)	0(0.0)
**Mother’s current marital status**									
Married*	4116(88.9)	3232(89.8)	2523(90.0)	1888(90.6)	1360(91.7)	897(91.3)	533(91.3)	310(92.8)	310(93.7)
Widowed	200(4.3)	159(4.4)	132(4.7)	108(5.2)	73(4.9)	53(5.4)	35(6.0)	13(3.9)	6(1.8)
Divorced	316(6.8)	209(5.8)	147(5.2)	88(4.2)	50(3.4)	33(3.4)	16(2.7)	11(3.3)	15(4.5)
**Place of delivery**									
Home*	609(57.6)	459(54.7)	408(59.5)	364(62.1)	305(65.0)	208(64.2)	131(63.3)	82(71.3)	84(70.6)
Health sector	449(42.4)	380(45.3)	278(40.5)	222(37.9)	164(35.0)	116(35.8)	76(36.7)	33(28.7)	35(29.4)
**Residence**									
Urban*	1136(24.5)	711(19.8)	454(16.2)	302(14.5)	217(14.6)	155(15.8)	95(16.3)	62(18.6)	66(19.9)
Rural	3496(75.5)	2889(80.3)	2348(83.8)	1782(85.5)	1266(85.4)	828(84.2)	489(83.7)	272(81.4)	265(80.1)
**Mother’s education level**									
No education*	2861(61.8)	2499(69.4)	2095(74.8)	1638(78.6)	1217(82.1)	814(82.8)	498(85.3)	279(83.5)	285(86.1)
Primary	1269(27.4)	860(23.9)	587(20.9)	393(18.9)	244(16.5)	156(15.9)	82(14.0)	52(15.6)	46(13.9)
Secondary and above	502(10.8)	241(6.7)	120(4.3)	53(2.5)	22(1.5)	13(1.3)	4(0.7)	3(0.9)	0(0.0)
**Husband/partner’s education level**									
Primary*	1269(71.)	860(78.1)	587(83.0)	393(88.1)	244(91.7)	156(92.3)	82(95.3)	52(94.5)	46(100.0)
Secondary and above	502(28.3)	241(21.9)	120(17.0)	53(11.9)	22(8.3)	13(7.7)	4(4.7)	3(5.5)	0(0.0)
**Region**									
Tigray*	385(8.3)	294(8.2)	225(8.0)	159(7.6)	105(7.1)	63(6.4)	28(4.8)	14(4.2)	13(3.9)
Afar	401(8.7)	312(8.7)	229(8.2)	167(8.0)	121(8.2)	85(8.6)	46(7.9)	26(7.8)	27(8.2)
Amhara	514(11.1)	407(11.3)	321(11.5)	225(10.8)	147(9.9)	95(9.7)	50(8.6)	25(7.5)	26(7.9)
Oromia	587(12.7)	476(13.2)	407(14.5)	316(15.2)	241(16.3)	174(17.7)	106(18.2)	70(21.0)	77(23.3)
Somali	387(8.4)	334 (9.3)	278(9.9)	233(11.2)	183(12.3)	128(13.0)	79(13.5)	56(16.8)	57(17.2)
Benishangul	420(9.1)	343(9.5)	278(9.9)	202(9.7)	151(10.2)	95(9.7)	58(9.9)	27(8.1)	29(8.8)
SNNPR	599(12.9)	505(14.0)	402(14.3)	318(15.3)	234(15.8)	155(15.8)	101(17.3)	53(15.9)	39(11.8)
Gambela	412(8.9)	325(9.0)	253(9.0)	183(8.8)	116(7.8)	64(6.5)	33(5.7)	12(5.7)	7(3.6)
Harari	361(7.8)	259(7.2)	184(6.6)	128(6.1)	86(5.8)	55(5.6)	33(5.7)	19(5.7)	21(6.3)
Addis Ababa	230(5.0)	113(3.1)	46(1.6)	21(1.0)	10(0.7)	4(0.4)	2(0.3)	0(0.0)	0(0.0)
Dire Dawa	336(7.3)	232(6.4)	179(6.4)	132(6.3)	89(6.0)	65(6.6)	48(8.2)	32(9.6)	35(10.6)
**Religion**									
Orthodox*	1524(32.9)	1122(31.2)	829(29.6)	561(26.9)	371(25.0)	224(22.8)	116(19.9)	56(16.8)	51(15.4)
Protestant	954(20.6)	753(20.9)	602(21.5)	461(22.1)	330(22.3)	215(21.9)	137(23.5)	70(21.0)	60(18.1)
Muslim	2052(44.3)	1644(45.7)	1304(46.5)	1008(48.4)	750(50.6)	521(53.0)	319(54.6)	201(60.2)	213(64.4)
Catholic,traditional +	102(2.2)	81(2.3)	67(2.4)	54(2.6)	32(2.2)	23(2.3)	12(2.1)	7(2.1)	7(2.1)
**Household size**									
1–4 (small)*	1238(26.7)	531(14.8)	300(10.7)	201(9.6)	131(8.8)	88(9.0)	52(8.9)	28(8.4)	19(5.7)
5–9 (medium)	3133(67.6)	2841(78.9)	2289(81.7)	1695(81.3)	1178(79.4)	741(75.4)	395(67.6)	216(64.7)	211(63.7)
10 and more (Large)	261(5.6)	228(6.3)	213(7.6)	188(9.0)	174(11.7)	154(15.7)	137(23.5)	90(26.9)	101(30.5)
**Household wealth index**									
Poorest*	1317 (28.4)	1106(30.7)	910(32.5)	703 (33.7)	529(35.7)	353(35.9)	210(36.0)	121(36.2)	115(34.7)
Poorer	806(17.4)	681(18.9)	565(20.2)	430(20.6)	296(20.0)	195(19.8)	114(19.5)	61(18.3)	57(17.2)
Middle	749(16.2)	614(17.1)	492(17.6)	373(17.9)	263(17.7)	180(18.3)	102(17.5)	58(17.4)	56(16.9)
Richer	720(15.5)	568(15.8)	453(16.2)	351(16.8)	249(16.8)	160(16.3)	100(17.1)	62(18.6)	72(21.8)
Richest	1040(22.5)	631(17.5)	382(13.6)	227(10.9)	146(9.8)	95(9.7)	58(9.9)	32(9.6)	31(9.4)
**Sex of child**									
Male	2426 (52.4)	1832(50.9)	1432(51.1)	1078(51.7)	759(51.2)	468(47.6)	287(49.1)	175(52.4)	167(50.5)
Female	2206 (47.6)	1768(49.1)	1370(48.9)	1006(48.3)	724(48.8)	515(52.6)	297(50.9)	159(47.6)	164(49.5)
**Child is alive**									
Yes*	4166 (89.9)	3272(90.9)	2570(91.7)	1916(91.9)	1341(90.4)	880(89.5)	524(89.7)	287(85.9)	274(82.8)
No	466 (10.1)	328(9.1)	232(8.3)	168(8.1)	142(9.6)	103(10.5)	60(10.3)	47(14.1)	57(17.2)

Key: * = *p*-value < 0.05

As compared to mothers who are living in the urban area and home place of delivery, the proportion of mothers in the rural area and health sector place of delivery was higher respectively at each recurrent birth event. Among mothers who have first and ninth recurrent birth events, 75.5% and 80.1% of them respectively were rural residents while 57.6% and 70.6% of them respectively delivered at home. The highest prevalence of first recurrent births (44.3%) was obtained from Muslim women. Frequently, as compared to mothers whose education levels were primary and above, illiterate mothers have a higher proportion per each recurrent birth event. The proportion of children's death at each recurrent birth was greater than 8.1% of the total children in the corresponding recurrent birth. As compared to poorer, middle, richer, and richest households, the proportion of mothers from the poorest household was the highest at each recurrent birth event. For instance, in the first recurrent birth, the proportion of poorest mothers (28.4%) was higher compared to poorer (17.4%), middle (16.2%), richer (15.5%), and richest (22.5%) mothers. Mothers from the SNNPR region had a higher proportion of first (12.9%) and second (14.0%) recurrent birth while the proportion of mothers in the Oromia region was higher from third (14.5%) to ninth (23.3%) recurrent birth.

**Fig. 3:**
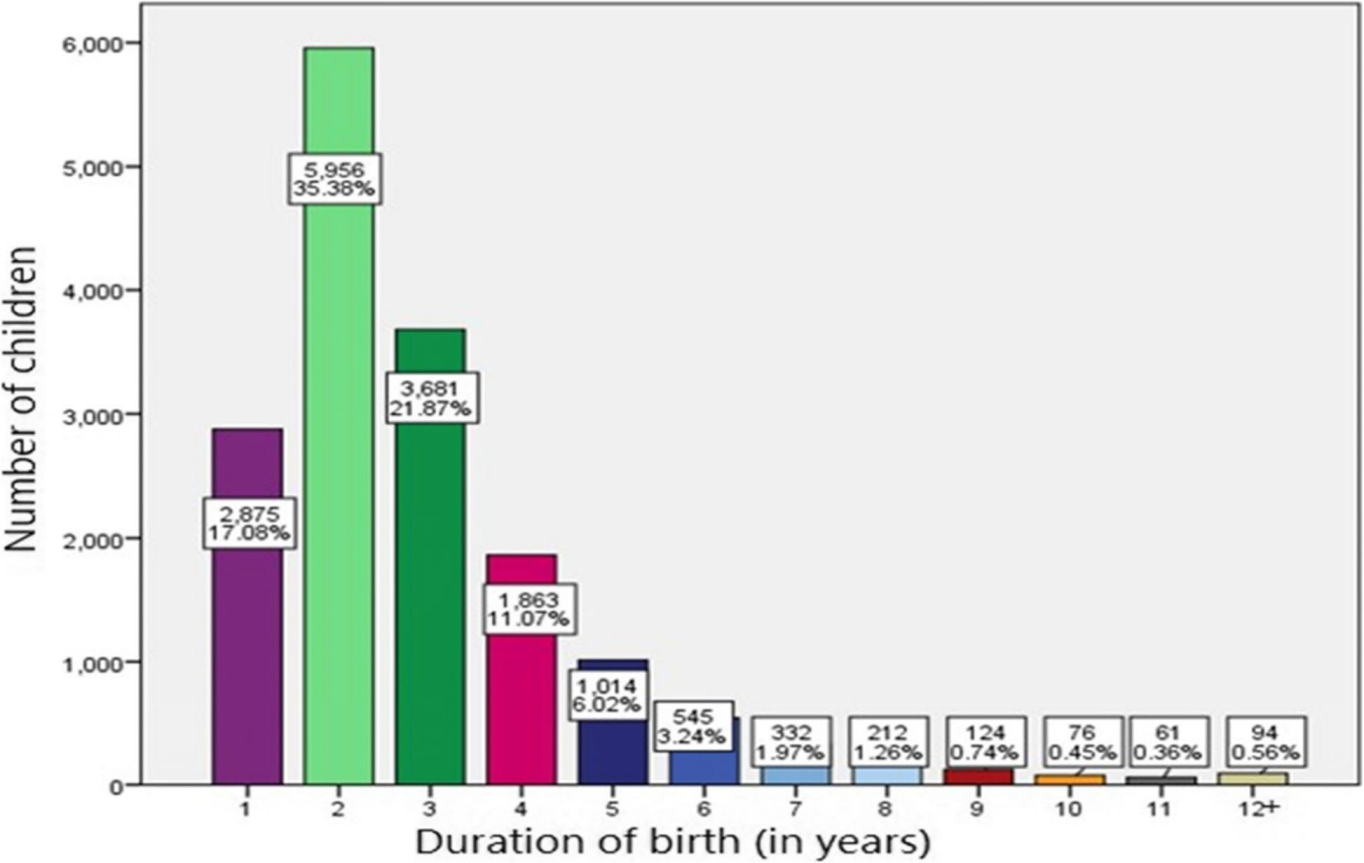
Bar plots of the number of children per duration of birth (in years)

Figure 3 shows gaps between successive childbirth (duration of birth) in terms of years. The majority of the children were born within two years (35.38%), three years (21.87%), and one year (17.08%) years interval consecutively in rank. This indicates the third-highest proportion of children were born within one year of the birth interval. The highest proportion of children within two years interval was also noticed in Table 2, the median follows up time for most of the recurrent birth events was two years.

### Parameter estimation

The estimated effects of mothers, household, and child characteristics on recurrent birth events using the AG model, PWT-TT model, PWP-GT model, and frailty model were fitted, see Table 5. The estimated hazard ratio (HR) and its 95% CI for each characteristic were computed within each model. The estimated effects of characteristics on recurrent birth were slightly different for different models as the identity of the models are different. The dependency between characteristics obtained from mothers, households, or children and recurrent birth events was measured using a hazard ratio (HR). The estimated 95% CI of HR of a character consists of one indicates an insignificant dependency between the corresponding characteristics and recurrent birth and the reverse is true if the 95% CI of HR does not include one.

**Table 5:** Hazard ratio (HR) estimate independent variables using AG, PWP-TT, PWP-GT, Frailty Model

Characteristics	AG model	PWP-TT model	PWP-GT model	Frailty Model
	HR (95% CI)	HR (95% CI)	HR (95% CI)	HR (95% CI)
**Age of mothers at first birth**				
< 20 (ref.)	1.000	1.000	1.000	1.000
20–34	**0.713 (0.614,0.828)**	1.052(0.933,1.188)	**0.852(0.733,0.992)**	**0.730(0.620,0.828)**
35–49	**0.050 (0.006,0.090)**	0.384(0.053,2.794)	0.189(0.00,9.024)	**0.725(0.626,0.849)**
**Mother’s current marital status**				
Married(ref.)	1.000	1.000	1.000	1.000
Widowed	1.140(0.834,1.540)	1.059(0.822,1.363)	0.911(0.658,1.263)	0.977(0.705,1.356)
Divorced	1.440(0.720,2.860)	1.063(0.613,1.843)	1.243(0.692,2.234)	1.527(0.829,2.816)
**Place of delivery**				
Home(ref.)	1.000	1.000	1.000	1.000
Health sector	**0.886(0.768,0.997)**	0.712(0.907, 1.165)	0.970(0.837,1.223)	0.935(0.805,1.087)
**Residence**				
Urban(ref.)	1.000	1.000	1.000	1.000
Rural	1.020(0.836,1.230)	0.939(0.792,1.113)	**0.885(0.706,0.995)**	1.011(0.824,1.240)
**Mother’s education level**				
No education(ref.)	1.000	1.000	1.000	1.000
Primary	**1.210(1.010,1.460)**	1.047(0.913,1.200)	1.049(0.885,1.245)	0.853(0.713,1.019)
Secondary and above	0.989(0.730,1.340)	0.960(0.769,1.204)	0.899(0.752, 1.076)	0.892(0.710,1.130)
**Husband/partner’s education level**				
Primary(ref.)	1.000	1.000	1.000	1.000
Secondary and above	1.200(0.945, 1.520)	0.953(0.795,1.142)	0.977 (0.825, 1.157)	1.210(0.976,1.501)
**Region**				
Tigray(ref.)	1.000	1.000	1.000	1.000
Afar	**0.657 (0.438,0.987)**	1.061(0.768,1.467)	0.960(0.640,1.442)	0.717(0.475,1.083)
Amhara	0.768(0.541,1.090)	1.147(0.858,1.532)	0.998(0.692,1.437)	0.848(0.585,1.230)
Oromia	**0.736(0.543,0.997)**	1.074(0.830,1.389)	0.985(0.720,1.348)	0.782(0.567,1.080)
Somali	0.850(0.598,1.210)	1.091(0.776,1.534)	0.888(0.593,1.329)	0.814(0.542,1.222)
Benishangul	0.833(0.614,1.130)	1.056(0.808,1.380)	0.957(0.694,1.320)	0.799(0.576,1.108)
SNNPR	0.942(0.698,1.270)	1.145(0.882,1.488)	1.296(0.943,1.781)	0.982(0.713,1.353)
Gambela	0.771(0.554,1.070)	0.995(0.759,1.304)	0.904(0.650,1.258)	0.753(0.537,1.055)
Harari	0.775(0.554,1.090)	1.013(0.772,1.330)	1.050(0.750,1.469)	0.780(0.555,1.100)
Addis Ababa	0.716(0.490,1.050)	1.055(0.802,1.386)	1.029(0.725,1.461)	0.749(0.521,1.076)
Dire Dawa	0.820(0.567,1.190)	1.136(0.848,1.521)	1.066(0.739,1.538)	0.757(0.521,1.099)
**Religion**				
Orthodox(ref.)	1.000	1.000	1.000	1.000
Protestant	1.230(0.971,1.560)	1.003(0.837,1.203)	1.078(0.857,1.358)	1.141(0.904,1.441)
Muslim	**1.260(1.010,1.580)**	1.016(0.861,1.199)	1.056(0.855,1.305)	1.210(0.976,1.501)
Catholic,traditional +	1.03(0.586,1.810)	0.834(0.553,1.257)	0.864(0.528,1.413)	1.047(0.638,1.719)
**Household size**				
1–4 (small)(ref.)	1.000	1.000	1.000	1.000
5–9 (medium)	**3.110(2.49,3.880)**	**1.914(1.539,2.381)**	**1.804(1.455,2.237)**	**3.183(2.605,3.889)**
10 and more (Large)	**2.660(1.870,3.770)**	**1.813(1.293,2.541)**	**1.880(1.352,2.613)**	**2.718(1.960,3.770)**
**Household wealth index**				
Poorest(ref.)	1.000	1.000	1.000	1.000
Poorer	0.957(0.778,1.180)	0.953(0.795,1.143)	0.838(0.682,1.030)	0.904(0.732,1.116)
Middle	0.845(0.683,1.050)	**0.827(0.688,0.994)**	**0.675(0.546,0.834)**	**0.776(0.625,0.965)**
Richer	0.950(0.751,1.200)	1.068(0.878,1.298)	0.859(0.683,1.081)	0.892(0.705,1.128)
Richest	1.070(0.830,1.380)	0.962(0.767,1.205)	0.941(0.730,1.212)	1.067(0.822,1.385)
**Sex of child**				
Male(ref.)	1.000	1.000	1.000	1.000
Female	**0.853(0.776,0.938)**	**0.836(0.755,0.926)**	**0.835(0.739,0.944)**	0.911(0.805,1.031)
**Child is alive**				
Yes(ref.)	1.000	1.000	1.000	1.000
No	**0.783(0.631,0.971)**	**0.705(0.533,0.934)**	**0.673(0.514,0.881**)	**0.681(0.522,0.889)**
Frailty Variance				**0.148**

Key:- *HR *Hazard Ratio, *CI* Confidence Interval, *Ref.* Reference

### AG model

Using the AG model, the age of mothers at first birth, place of delivery, mothers' education level, region, religion, household size, sex of the child, and child mortality have significant effects on the recurrent birth. The estimated hazard of recurrent birth of mothers who have a primary education level was 1.210 times the estimated hazard of recurrent birth of mothers who have no education (HR:1.210; 95%CI: 1.010, 1.460). The estimated hazard of recurrent birth of mothers whose ages were between 20 and 34 years old was lower by 28.7% than the estimated hazard of recurrent birth of mothers whose ages were less than 20 years old (HR:0.713; 95% CI: 0.614, 0.828). The hazard ratio of recurrent birth of the hazard of Muslim to Orthodox households was 1.260 indicates that the estimated hazard of recurrent birth in Muslim households was higher by 26.0% than the estimated hazard of recurrent birth in Orthodox households. Mothers whose place of delivery was in the health sector had a lower estimated hazard of recurrent birth as compared to mothers whose place of delivery was at home.

### PWP-TT model, PWP-GT model, and Frailty model

The parameter estimates obtained from PWP-TT, PWP-GT, and Frailty models were closer to each other for characteristics of household size, household wealth index, and child mortality. The household size, household wealth index, and child mortality have a significant effect on recurrent birth. The estimated hazard of recurrent birth in households that had a middle wealth index was 0.776 times the estimated hazard of recurrent birth in a household with the poorest wealth index. This refers to the estimated hazard of recurrent birth in the household that had a middle wealth index was lower by 22.4% of the risk of recurrent birth in the household who have poorest wealth index (HR:0.776; 95% CI: 0.625,0.965) which indicates that the poorest household was more likely to have higher recurrent birth as compared to the household with middle wealth index. The estimated hazard of recurrent birth of mothers who haven’t died children was 0.673 times the estimated hazard of recurrent birth of mothers who have died children (HR:0.673; 95%CI:0.514,0.881). This means the estimated hazard of recurrent birth of mothers who haven’t died children was lower by 32.7% of the estimated hazard of recurrent birth of mothers who have died children. Households with medium (5–9) and large (10 and more) family sizes had a higher risk of recurring birth as the estimated hazard ratio was greater than one (HR: 1.914; 95%CI:1.539,2.381 and HR:1.880; 95% CI:1.352,2.613, respectively).

Results from the PWP-TT model and PWP-GT model point out the sex of child have a significant effect on the recurrent birth in which mothers who have female children had a lower risk of recurrent birth as compared to mothers who have male children. The estimated hazard of mothers who have female children was 0.836 (HR:0.836; 95%CI = 0.755,0.926) times the estimated hazard of mothers who have male children and it is almost the same in the case of the PWP-TT model and PWPGT model. Based on the PWP-GT model, the effect of place of residence and age of mothers at first birth was not null (HR:0.885; 95%CI: 0.706,0.995 and HR:0.852; 95% CI: 0.733, 0.992). Besides, results from the frailty model was also shows the age of mothers at first birth had a significant effect on recurrent birth in which the risk of recurrent birth of mothers aged 20–34 and 35–49 years was lower by 27.0% (HR:0.730; 95% CI: 0.620, 0.828) and 27.5% (HR:0.725; 95%CI:0.626, 0.849) respectively of the estimated risk of recurrent birth of mothers whose age less than 20 years old.

## Discussion

In this study, the exploratory data analysis of recurrent birth events and important mothers, household, and child characteristics that are associated with recurrent birth in Ethiopia were identified using the extended cox model such as the AG model, PWP-TT model, PWP-GT, and frailty model. The median follow-up time for 3rd, 4th, 5th, 6th, 7th, 8th, 9th, 10th, 11th, 12th, and 13th recurrent birth was two years. The minimum duration of birth (0.95 years) from 4^th^, 12^th^, and 14^th^ recurrent birth events indicate a women successive birth within a gap of less than a year. Over 51 percent of women had greater than or equal to three recurrent births. The term recurrent birth was defined as two or more successive deliveries of mothers [5]. As a result, mothers who have at least one recurrent birth were considered in this study while the first delivery of mothers was considered as the origin of the follow-up.

Ethiopia is one of the countries with a high fertility rate in Africa [4]. In this study, from 4676 mothers a total of 16833 children were obtained indicating that a mother had more than three children on average. The maximum recurrent birth of mothers was fourteen and the minimum birth interval was 0.95 years. The median recurrent birth time interval between subsequent birth events was two years which was greater than the subsequent birth intervals (22 months) in Uganda [5]. The duration of recurrent birth of over one-third of mothers was approximately two years whereas over 15% of mothers considered in this study had one year of a duration birth. In contrast, a study finding based on 2016 EDHS reported that the median inter-birth interval is over three years [4]. The birth interval of Ethiopia was higher compared to Tanzania (19.0%), Nigeria (23.0%), and Kenya (18.0%) [5]. Thus, the recurrent birth interval of Ethiopian mothers (24 months) not met the minimum birth interval set by WHO, a minimum of 33 months between two successive live births is recommended. This might be because they have a low practice of contraceptive use due to cultural as well as religious issues. This contributes to adverse effects on the child as well as maternal health [5]. Among numerous short birth intervals, adverse effects on child stunting, underweight and wasting [16, 17] are the most common despite it is also substantially associated with the household wealth index [18].

Place of delivery and residence, age of mothers at first birth, mother’s current marital status, mothers and husband/partner education level, region, religion, household size, household wealth index, and child mortality was independently associated with recurrent birth events. The highest recurrent birth occurred during the age less than twenty years old of mothers at first birth as compared to mothers whose age older at first birth. This is in line with findings in [19, 20] stating the increment infertility rate is because of first marriage at an early age and low contraceptive utilization practice. Over sixty percent of Ethiopian women aged 20–49 got married before the age of 18 [20]. Besides, women at a younger age would not have mature awareness about the consequence of early marriage and birth as they were enforced by the societies. Mothers who have no education and mothers with the poorest wealth index had a higher proportion of first, second, third, and fourth recurrent birth compared to the corresponding counter group, i.e. educated and rich mothers respectively. There is also recurrent birth difference across regions in which the highest and lowest recurrent birth was noticed from SNNPR and Addis Ababa respectively which was consistent with a study in [19], which reported that fertility trends and levels are different between regions. This might be because the attitudes of people across the region in terms of fertility in particular related to religion are different. For instance, in Somalia and Harari region over nineteen percent of the people follow the Muslim religion and have a poor habit of practicing contraceptive use to maintain their religious doctrine.

In the AG model, the effects of mothers' education level, place of residence and place of delivery, age of mothers at first birth, region, household size, sex of the child, and child mortality had a significant effect on recurrent birth. As a lack of studies on recurrent birth events and associated factors, mostly our study findings were compared to a study related to birth intervals and recurrent preterm birth. The risk of recurrent birth of mothers whose ages were below twenty years old was higher as compared to mothers whose ages were 20–34 and 35–49 years old. This was in line with a study on early marriage in Ethiopia [20] depicted that early marriage of women was the major reason to have higher fertility and recurrent birth. This is because women at an early age have lack of maturity to plan the time when the subsequent child might occur and lack contraceptive practice that is isolated from cultural and religious interference [19, 20]. The region where mothers are living has also a substantial effect on recurrent birth in which the risk of recurrent birth in Afar and Oromia was lower by 34.3% and 26.4% respectively than the risk of recurrent birth of mothers in the Tigray region. These findings were supported by a study in [21] that revealed that there is a considerable birth difference across regions. A study reported in overall sub-Saharan Africa [22], Muslim mothers had a higher likelihood of recurrent birth as compared to non-Muslim mothers. This might be because non-muslim mothers have better contraceptive utilization practices as compared to Muslim mothers [22].

The estimated hazard of recurrent birth of mothers who have female children was lower by 11.12% than the estimated hazard of recurrent birth of mothers who have male children. In Ethiopia, commonly women who have a child of the same sex successively are eager to deliver a child of different sex, and hence the actual number of children in the family becomes higher than the desired number of children. It is also reported in a study in Uganda [5], that when women have more female children, the recurrent birth becomes increases in the quest for a male child and vice versa. This is also done sometimes when mothers have died children in the near past. Thus, mothers who had dead children were more likely to have recurrent birth compared to mothers whose child alive. A study in Zimbabwe, Kenya, Malawi, Lesotho, and Tanzania showed that the death of an index child increased the likelihood of a mother having another birth [22, 23].

Likewise in the AG model, household size, sex of the child, and child mortality had also a significant effect on recurrent birth using PWP-TT, PWP-GT model, and frailty model. Mothers from the poorest Wealth index had higher recurrent birth than mothers from the middle wealth index. This was consistent with analysis results obtained from 2018 NHDS [23] in Nigeria, which reported that the hazard of the birth interval of mothers was lower for women whose economic status was middle and rich compared to mothers whose economic status was poor. The estimated risk of recurrent birth of mothers from the rural area was lower than 11.5% of the estimated risk of recurrent birth of mothers from the urban area and it is supported by [23], the significant recurrent birth not only between urban and rural areas but also between zones. In contrast, a study finding in [21] reported that as compared to single mothers, the mothers whose marital status was divorced, widowed/separated, and married had a longer birth interval, and the effect of mothers' current marital status on recurrent birth events was null.

### Limitation of the study

The findings of this study are limited to data obtained from a survey EDHS 2019. It would be better if a further study can be conducted on previous surveys EDHS2016, EDHS 2011, etc. to assess the progress in detail. Many different studies related to recurrent birth have used different predictor variables and some of them arrived at different results. Therefore we recommended that further studies on this topic include other important covariates that were not included in this study which could have a potential confounding effect.

## Conclusion

The recurrent birth interval of Ethiopian mothers was less than the minimum birth interval set by WHO, a minimum of 33 months between two successive live births is recommended. The highest recurrent birth occurred during the age less than twenty years old of mothers at first birth as compared to mothers whose age was older at first birth. The effects of mothers' education level, place of residence and place of delivery, age of mothers at first birth, region, household size, sex of the child, and child mortality had a significant effect on recurrent birth. Besides, mothers from the poorest economic status and the rural area had higher recurrent birth than mothers from middle economic status and urban areas, respectively. We authors would like to recommend communities, governmental and non-governmental stakeholders consider the associated factors of frequent recurrence of birth noticed in this study. The age of 70.5% of mothers in this study was less than twenty years old at their first birth. Thus, we would also like to recommend women start birth while they got mature in age to reduce frequent recurrent birth and its corresponding adverse effects. 

## Data Availability

The data that the authors used to produce this manuscript are available upon reasonable request from demographic and health survey (DHS) cite www.dhsprogram.com. The DHS Program is authorized to distribute, at no cost, unrestricted survey data files for legitimate academic research. Registration is required for access to data.

## Abbreviations

EDHS: Ethiopian demographic and health survey
NDHS: Nigerian demographic and health survey
HR: Hazard ratio
CI: Confidence interval
SNNPR: South Nations Nationalities and Peoples Representatives
WHO: World health organization
